# Physical activity monitoring in Alzheimer’s disease during sport interventions: a multi-methodological perspective

**DOI:** 10.3389/fneur.2023.1195694

**Published:** 2023-09-22

**Authors:** Shari David, Christian Hohenfeld, Luisa Haberl, Jennifer Pahl, Ana S. Costa, Axel Kilders, Frank Hildebrand, Jörg B. Schulz, Kathrin Reetz, Alexa Haeger

**Affiliations:** ^1^Department of Neurology, RWTH Aachen University, Aachen, Germany; ^2^Institute of Neuroscience and Medicine (INM-11), Forschungszentrum Jülich GmbH, Jülich, Germany; ^3^JARA-BRAIN Institute, Jülich, Germany; ^4^Department of Physiotherapy, RWTH Aachen University, Aachen, Germany; ^5^Department of Orthopaedic, Trauma and Reconstructive Surgery, RWTH Aachen University, Aachen, Germany

**Keywords:** physical activity intervention, exercise, fitness, Alzheimer’s disease, dementia, wearables, mobile health, fitness tracker

## Abstract

**Introduction:**

Assessment methods for physical activity and fitness are of upmost importance due to the possible beneficial effect of physical conditioning on neurodegenerative diseases. The implementation of these methods can be challenging when examining elderly or cognitively impaired participants. In the presented study, we compared three different assessment methods for physical activity from the Dementia-MOVE trial, a 6-months intervention study on physical activity in Alzheimer’s disease. The aim was to determine the comparability of physical activity assessments in elderly participants with cognitive impairment due to Alzheimer’s disease.

**Material or methods:**

38 participants (mean age 70 ± 7 years) with early-stage Alzheimer’s disease (mean MoCA 18.84 ± 4.87) were assessed with (1) fitness trackers for an average of 12 (± 6) days, (2) a written diary on daily activities and (3) a questionnaire on physical activity at three intervention timepoints. For comparison purposes, we present a transformation and harmonization method of the physical assessment output parameters: Metabolic equivalent of task (MET) scores, activity intensity minutes, calorie expenditure and moderate-to-vigorous physical activity (MVPA) scores were derived from all three modalities. The resulting parameters were compared for absolute differences, correlation, and their influence by possible mediating factors such as cognitive state and markers from cerebrospinal fluid.

**Results:**

Participants showed high acceptance and compliance to all three assessment methods. MET scores and MVPA from fitness trackers and diaries showed high overlap, whilst results from the questionnaire suggest that participants tended to overestimate their physical activity in the long-term retrospective assessment. All activity parameters were independent of the tested Alzheimer’s disease parameters, showing that not only fitness trackers, but also diaries can be successfully applied for physical activity assessment in a sample affected by early-stage Alzheimer’s disease.

**Discussion:**

Our results show that fitness trackers and physical activity diaries have the highest robustness, leading to a highly comparable estimation of physical activity in people with Alzheimer’s disease. As assessed parameters, it is recommendable to focus on MET, MVPA and on accelerometric sensor data such as step count, and less on activity calories and different activity intensities which are dependent on different variables and point to a lower reliability.

## Introduction

The rising incidence of dementia, and especially Alzheimer’s disease (AD) as the most common form, represents a major public health challenge, imposing not only on the people affected, but also on our entire society and health system (1). Next to pharmacological therapies, intervention studies investigating the effects of modifiable risk factors in delaying or preventing dementia, have suggested that improved physical activity and cardiorespiratory fitness could slow down cognitive decline and positively influence brain pathology in neurodegeneration (2–5), either as a stand-alone or as add-on on primary and secondary therapies (6–9). The individual contribution to brain pathology and neurodegeneration on the general physical activity level, on the one hand, and cardiorespiratory fitness, on the other hand, assessed, e.g., by the maximal oxygen uptake (VO_2_max), is still not clear and rarely differentiated in intervention studies in AD (2).

In the context of this type of intervention studies, tracking physical activity and cardiorespiratory fitness is a major challenge, especially in elderly and cognitively impaired people. Such challenges are related to issues with patient’s acceptability and compliance, increased susceptibility to biases (e.g., social desirability bias), but also lack of reference values that adjust for confounders, such as age and motor disability (10, 11). Beyond classical approaches for monitoring physical activity that use self-report, such as diaries and standardized questionnaires, focus is now set on a new field using wearable and affordable fitness trackers and mobile health devices (12, 13). Besides recording physical activity data, this type of devices also offer additional options to track sleep parameters enabling and objectifying the investigation of neuropsychiatric health and impairment of the autonomic nervous system in AD and therefore link to behavioral symptoms in AD pathology (14). Still, intervention studies of physical activity and cardiorespiratory fitness in AD that apply these types of devices are scarce.

The Dementia-MOVE study is a six-months randomized-controlled intervention study to investigate the effect of physical activity and cardiorespiratory fitness on AD progression. Three different methods were used for the assessment of physical activity and individual cardiorespiratory fitness levels: (1) a wearable fitness tracker for the recording of physical activity and sleep, (2) a written diary, in which participants recorded their daily physical activities and sleep duration and (3) a standardized questionnaire on physical activity in the previous month. In the present study, we compare these three different fitness / activity tracking methods in terms of feasibility and reliability in a group of elderly cognitively impaired people in the context of an intervention study. Our aim is to clarify how accurate and feasible the assessment of physical activity and fitness in people with AD is, and in such a way to contribute to increased quality standards for upcoming intervention studies.

## Methods

### The Dementia-MOVE multicomponent intervention program in Alzheimer’s disease

The Dementia-MOVE study (Multi-Objective Validation of Exercise in Dementia) is a six-months randomized-controlled clinical trial with two arms, including a group participating in an exercise intervention program compared to a group participating in a pure psychoeducational program (control group). A total of 46 people diagnosed with prodromal or mild AD according to NIA-AA Research Framework criteria (15) were included in the trial. People aged between 50 and 80 years that were cognitively and physically able to participate in the whole study program were considered eligible. After baseline assessments (timepoint T1), participants were randomized into either the exercise intervention group (*n* = 26) or a control group (*n* = 20), using the WINPEPI software (16). Randomization was performed blinded for clinical information, balancing for age and fitness level (VO_2_max or 6-min walk distance). Further outcome assessments were collected after three (timepoint T2) and 6 months (timepoint T3). From the outcome assessments, in this study we focused on fitness assessment using the 6-min walk test (6-MWT) (17) and estimated VO_2_max determined by an ergometer protocol (18), as well as global cognitive performance using the Montreal Cognitive Assessment (MoCA) screening test (19), the Clinical Dementia Rating (CDR) (20) and neuropsychiatric symptoms, namely the Hospital Anxiety and Depression Scale (HADS) (21). The exercise intervention included both a weekly training program guided by physiotherapists, as well as a home-based training program. For the home-based training, participants were instructed to undergo physical training at home for at least 30 min a week, with moderate exertion according to the Borg rating of perceived exertion (RPE) scale adapted to people with dementia, and received instructions for stretching and toning exercises, which they should also perform twice a week for 15 min (22, 23). The control group was instructed to perform their physical activity as usual and received monthly psychoeducational sessions, in which the intervention group also participated. During the lockdown period due to the SARS-CoV2 pandemic, which affected a subgroup of participants, all intervention was changed to a home-based program and narrowly monitored by regular phone calls and reminders.

The study and all protocol changes were approved by the local ethics committee (EK 306/18) and performed according to the latest Declaration of Helsinki. All participants gave written informed consent to study participation and protocol changes. The trial was registered at ClinicalTrials.gov (NCT03939286). The targeted sample size was initially set to 50 people, based on power analyses and previous intervention studies with physical activity. The detailed study protocol is described in Haeger et al. (24).

For the current analyses on activity monitoring, we included participants with a complete set of data. This implied that the recorded days of the fitness tracker had matching diary entries and excluded participants who dropped-out during the course of the study. Thus, the data presented here arises from a final sample of 38 participants, regardless of group randomization assignment.

### Assessment of physical activity

#### Assessment of physical activity via wearable fitness trackers

We used a commercially available wrist-worn fitness tracker (Fitbit Charge 2^®^) to determine activity levels (step counts, activity intensity and frequency). We created an anonymized Fitbit account for each participant, including individual relevant information (age, gender, weight and height). Participants used the devices for an average of 14 days around the T2 timepoint. They were instructed to wear the tracker also at night and to take it off only for showering and recharging in case of battery depletion. Since elderly and cognitively impaired study participants not always use smartphone devices and are therefore not necessarily able to regularly synchronize their fitness trackers, the devices were distributed on a normally weekly to biweekly basis. Intermittently, the devices were collected, synchronized by the study team and then handed out again. Following this strategy, there was a low efficiency due to overwriting of the device’s internal memory when time intervals between account synchronizations were too long. To generate as many physical activity information as possible, we then moved to regular synchronization at home at least every 3 days by instructing the participants and their caregivers. In case no smartphone was available, the trackers were synchronized via the first described method, so the possession of a smartphone was no eligibility criterion for study participation. Data generated by the device included the walking distance, step count, floors count, active minutes (divided in three rates from light to moderate and vigorous intensity), as well as activity calorie and total calorie consumption.

#### Assessment of physical activity via a written diary

As a second method, a paper-based physical activity written diary was distributed to participants, together with an information and instructional letter. By telephone, participants and their caregivers were verbally instructed about the correct usage of the diary. The diary took the physical form of a folder with removable sheets, with each page offering a weekly overview, with fields to enter the type and duration of daily activities, physical complaints during exercise, subjective physical exertion estimated via the Borg RPE scale (22), hours of sleep, medication taken, weight (measured once per week), and additional comments, such as current health complaints. All participants, independent of group randomization assignment, completed the diary. For the intervention group, rows to document each of the weekly training sessions were also available. An exemplary diary page translated into English can be found in Supplementary Table S1. To minimize the risk of missing data due to loss of single diary pages, completed pages were collected as often as possible, for example during the monthly psychoeducational lectures, study visits, as well as the supervised trainings for the intervention group. The collected pages were then checked by the study team and participants and their relatives were again instructed, in the case of incompleteness or inaccuracy to avoid recall issues. As the people included in the study had a CDR score of 0.5 to 1 on average indicating a relatively preserved autonomy, keeping a diary was possible. The presence of a caregiver was therefore not obligatory, however, only one participant in our study sample did not have relatives (either a partner or children) for support at home. The diary had to be filled out during the whole 26 weeks of study participation. For nine participants, the T3 visit had to be postponed for 9 weeks due to the SARS-CoV-2 lockdown. The intervention protocol was kept the same during this period and only changed to a home-based program with similar exercises. The participants were instructed to continue keeping the diary for this additional time to keep track of their physical activity, until the final outcome assessments were feasible.

#### Assessment of physical activity via the PAQ50+ questionnaire

The PAQ50+ is a structured questionnaire for recording physical activity, especially designed for people over the age of 50 years (25). The PAQ50+ is based on the Yale Physical Activity Surveys and the Physical Activity Scale for the Elderly and measures how much time a person has spent on various activities over the past 4 weeks, in terms of hours per week (26, 27). It has been shown to be reliable in estimating both total physical activity and energy expenditure (28). It offers space to enter the time spent on 37 predefined activities from various categories such as housework, gardening, leisure time, sport, and occupation as well as further individual activities. Each activity determines a certain metabolic equivalent of task (MET)-score, which leads in combination with the total duration of the activity and the weight of the person to the estimation of the energy expenditure. The total score of the PAQ50+ is calculated by the sum of all activities in kilocalories per week. The PAQ50+ was administered to participants at all timepoints of study participation (baseline T1, T2, T3).

#### Physical activity data processing and transformation

The primary aim of data transformation was to standardize the output of the assessment tools to enable a reliable comparison between the different methods. Data of all three methods were processed based on MET scores. One MET is equal to the energy cost of sitting quietly and represents an estimation of the degree of energy expenditure of individual activities put in relation (29). Using this method, activities were converted according to their intensity to MET-hours per day applying the following equation based on Ainsworth et al. (30):


(1) $$\mathrm{Daily}\;\mathrm{MET}=\overset{A}{\underset{n=1}{\sum }}\frac{t_{n}\times \mathrm{ME}T_{n}}{60}\text{,}$$

with *A* being the number of distinct daily activities performed, *t_n_* the time in minutes spent on activity *n*, weighted by its individual MET (*MET_n_*).

In general, all activities with a MET >3 were defined as physical activity, based on the recommendations for physical activity according to the American College of Sports Medicine (31), and specified in previous literature (30, 32, 33), as well as on the Fitbit website.^1^ The estimation of active minutes from the Fitbit device is based on MET scores to estimate the exercise intensity. The Fitbit therefore demands an activity >3 MET to be continuously performed for at least 10 min to be recorded (34). An additional daily MET score was calculated first converting the Fitbit walking distance (in kilometers) to a time unit (in hours) by dividing it by a standard pace of 4 km/h and then multiplying it with a MET of 3.5 for walking (30). For the subsequent analyses, the moderate and vigorous intensities of physical activity were used and their sum cumulated in a moderate-to-vigorous physical activity (MVPA) score, as these cover the generally recommended training intensity for physical activity (32). We further applied the activity calories given by the fitness tracker in the analyses.

To enable a conversion to MET scores from the written diary, all entries were initially digitized and standardized indicating the activity and the time spent in minutes to meet the calculation requirements for eq. (1) above. MET scores of entries originally specified in distance units were computed as follows:


(2) $$\mathrm{Daily}\;\mathrm{MET}=\overset{A}{\underset{n=1}{\sum }}\left(\frac{d_{n}\;}{p_{n}}\right)\;\times \mathrm{ME}T_{n}\text{,}$$

with *A* being the number of distinct daily activities performed, *d_n_* the distance covered during the activity *n* in kilometers, divided by a predefined low-to-moderate intense standard pace *p_n_* in kilometers per hour as to be expected in the elderly, and multiplied by its individual *MET_n_* (30).

Entries with no time or distance information given were set to 30 min in general and 15 min for stretching and toning tasks, as these were the given instructions on time-duration for these additional home-based activities in the intervention group (24). A daily MET score was calculated for each day as the sum of all activities performed following the equations above. However, low-effort activities with a MET <2 were priorly filtered out for interindividual homogenization of the entries (30). Analogously to the Fitbit output, active minutes were computed based on previously defined MET thresholds indicating moderate exercise with a MET of 3–5.9 and vigorous activity with a MET ≥6 (30, 32, 33). Furthermore, activity calories were calculated by multiplying the daily MET score with the participant’s weight in kilograms as instructed in the PAQ50+ questionnaire.

The processing of the PAQ50+ data was carried out analogously to the physical activity diary. As mentioned before, MET scores for the 37 activities in the PAQ50+ are already included in the questionnaire. MET scores of individual entries were complemented using the same publication as for the physical activity diary (30). To enable comparison between methods, an average daily score was calculated by dividing the original weekly PAQ50+ score by 7.

The data transformation was carried out automatically using a program written in the Python programming language version 3.9^2^ implementing the processing rules listed above. Out of all variables examined, MET and MVPA were prioritized being the presumably most informative parameters to assess the basic physical activity level. The subdivided activity minutes scores as well as activity calories were additionally analyzed to supply further information on each method’s accuracy and potential differences in the data transforming process.

All three methods were compared based on the total data acquired during the intervention period. A mean value of the T2 and T3 scores only was calculated for the PAQ50+ as the T1 assessment referred to the time-period before the intervention start. Fitbit and physical activity diary were additionally compared focusing only on the recorded days and their corresponding diary entries.

### Statistical analysis

All statistical analyses were performed using IBM SPSS Statistics 26 software.^3^ An explorative data analysis revealed a non-normally distributed data structure of activity monitoring data, therefore nonparametric tests were used for comparison. Absolute parameters of all three methods as mentioned above were first compared for differences using mean, standard deviation, median, median absolute deviation, and the Wilcoxon-Test. Correlation analysis was performed using Spearman’s rank correlation including 95% confidence intervals computed via BCa-Bootstrapping with 2,000 samples. Bland–Altman-Plots were built to further visualize the fluctuation range of diary and PAQ50+ MET data compared to the objective fitness tracker assessments (35, 36). To control for possible influencing factors, additional partial correlations were performed controlling for cerebrospinal fluid (CSF) parameters such as amyloid-β 1–42, amyloid-β 42/40-ratio, tau, and phospho-tau as markers of neurodegeneration in AD, the MoCA score, the HADS, and years of education. Zero-order correlations of each analysis were also examined for significant associations between the fitness parameters and the correcting variable, to further analyze a noticeable stronger deviation of the partial correlation coefficients from the uncorrected analysis. Note that the sample size for partial correlation analyses correcting for CSF markers is reduced to *n* = 35, and *n* = 34 for amyloid-β 42/40-ratio, respectively, since three participants did not have CSF diagnostics. Each analysis was Bonferroni-Holm corrected for multiple comparisons, with significant results reported at a corrected *p-*value <0.05.

## Results

### Basic characteristics of the study sample

Mean age of the study sample was 70 (± 7) years, including 13 women and 25 men. In the baseline fitness assessment, the participants reached an average VO_2_max of 29.59 (± 5.74) ml/kg/min and covered a mean distance of 538 (± 97) meters in the 6-Minute-Walk-Test. The average body mass index (BMI) was 24.32 (± 4.28) kg/m^2^. The average brief cognitive screening MoCA score was 18.84 (± 4.87), the CDR mean score was 0.75 (± 0.38), marking a mild disease stage (20). A description of the included sample is given in Table 1.

**Table 1:** Basic characteristics of the analyzed sample (*n* = 38).

Sample characteristics	*N*	*M*	SD	Min	Max
Age (years)	38	69.63	7.32	50	80
Sex (m/f)	38	25/13			
Education (years)	38	13.34	3.6	5	21
CDR	38	0.75	0.38	0	2
CSF Amyloid-β 1–42 (pg/ml)^a^	35	414.97	170.63	135	841
CSF Amyloid-β 42/40-ratio^a^	34	0.42	0.2	0.18	1.3
CSF Tau (pg/ml)^a^	35	611.69	323.32	135	1,285
CSF Phospho-Tau (pg/ml)^a^	35	85.20	35.39	24	191
MoCA	38	18.84	4.87	10	28
HADS	38	7.92	6.54	0	25
VO_2_max (ml/min/kg)	38	29.59	5.74	16.55	45.93
6-MWT (m)	38	537.74	97.38	331.6	781
BMI (kg/m^2^)	38	24.32	4.28	18.71	41.77

a Lab-specific AD-relevant cut-off values for cerebrospinal fluid parameters are as follows: amyloid-β 1–42 < 450 pg/ml, amyloid-β 42/40-ratio < 0.5, tau >450 pg/ml, phospho-tau >61 pg/ml, analysis being performed in the Neurochemical Laboratory at the University of Göttingen (37, 38).

Overview of the characteristics of the presented study sample: Sample size *N*, mean values *M*, standard deviations SD and range (Min, Max values) are presented. CDR, Clinical Dementia Rating; MoCA, Montreal Cognitive Assessment; HADS, Hospital Anxiety and Depression Scale; VO_2_max, maximal oxygen uptake (in ml/kg/min); 6-MWT, 6-Minute Walk Test (in meters); BMI, body mass index (in kg/m2); CSF, cerebrospinal fluid.

### Adherence to physical activity acquisitions

The Fitbit tracker was distributed to the participants for approximately 14 days, providing an average of 12 (± 6) completely registered days. The compliance for wearing the fitness trackers was 100%. For the written diary, calculation of the compliance refers to 26 weeks of intervention and, thus, 182 editable daily columns, which were accepted as completed, if at least one row per day was filled out by the participant. According to these considerations, mean diary compliance was altogether high with 87% (± 20%). The PAQ50+ questionnaire was filled out by 100% of the 38 participants included in this analysis at all three acquisition timepoints.

### Comparison of physical activity assessment methods

First, we compared mean data from matching fitness tracker recording days and corresponding diary entries (see Table 2). Mean MET (5.73 ± 2.67 vs. 5.12 ± 4.41) as well as MVPA values (66.31 ± 49.33 vs. 63.47 ± 59.16) were not statistically different (for MET: *z* = −1.47, *p* = 0.71; for MVPA: *z* = −0.76, *p* = 0.45), revealing a high association between fitness tracking and subjective estimation of activity noted by the participants in the diaries. However, when differentiating between minutes spent on moderate and vigorous activity, data from the tracker and the diary were discrepant (moderate intensity, *z* = −1.25, *p* = 0.63; vigorous intensity, *z* = −4.07, *p* = <0.001), and suggesting differences in the subdivision algorithm between both methods. When comparing the registered activity from the fitness trackers with the activity noted in the diaries during the whole intervention period, MET and MVPA were similar, pointing to a high rate of transferability of the activity results of the registered fitness trackers period to the whole period of the intervention (MET, *z* = −1.39, *p* = 0.66; MVPA, *z* = −0.78, *p* = 0.88).

**Table 2:** Descriptive statistics of the three applied physical activity assessment methods.

Methods	Steps	MET score	Moderate activity (min)	Vigorous activity (min)	MVPA (min)	Activity calories (kcal)
Fitbit	9,262 (4478) *8,222 (2373)*	5.7 (2.7) *4.9 (1.5)*	32.2 (28.4) *22.2 (10.5)*	34.1 (27.6) *30.5 (15.3)*	66.3 (49.3) *51.2 (27)*	1,114 (447) *1,042 (332)*
Diary		5.1 (4.4) *3.7 (1.9)*	51.4 (60.4) *29 (19.2)*	12.1 (14.5) *6.7 (5.4)*	63.5 (59.2) *41.2 (20.7)*	365 (349) *286 (143)*
PAQ50+		16.4 (8.5) *16.7 (6.2)*	126.7 (74.6) *115.7 (41.8)*	27.5 (30.5) *18.2 (10.7)*	154.2 (83) *139.3 (50.4)*	1,135 (656) *1,042 (362)*

Daily mean values and standard deviations of Fitbit, diary and PAQ50+ are presented, as well as median values and median absolute deviations in italics. Indicated values of Fitbit and diary refer to Fitbit recording days with matching diary entries only. For the PAQ50+ mean values of the T2 and T3 acquisitions were used. MET, metabolic equivalent of task; MVPA, moderate-to-vigorous physical activity (in minutes).

Furthermore, we compared results of the PAQ50+ questionnaire with the fitness trackers and the diaries. Therefore, activity derived from the PAQ50+ at timepoints T2 and T3 was averaged, since only activity during the intervention period was of interest for this analysis. We noticed that even though the questionnaire was similarly subjective compared to the diary assessment, activity reported in the PAQ50+ was higher (MET, 16.42 ± 8.53; MVPA 154.21 ± 83) compared to the fitness tracker (MET, *z* = −5.33, *p* = <0.001; MVPA, *z* = −4.92, *p* = <0.001) and the diary (MET, *z* = −5.2, *p* = <0.001; MVPA, *z* = −5.16, *p* = <0.001). A difference in all three methods was also reflected in the activity calorie consumption (Fitbit, 1,116 ± 447; diary, 375 ± 317; PAQ50+, 1,135 ± 656).

**Figure 1:**
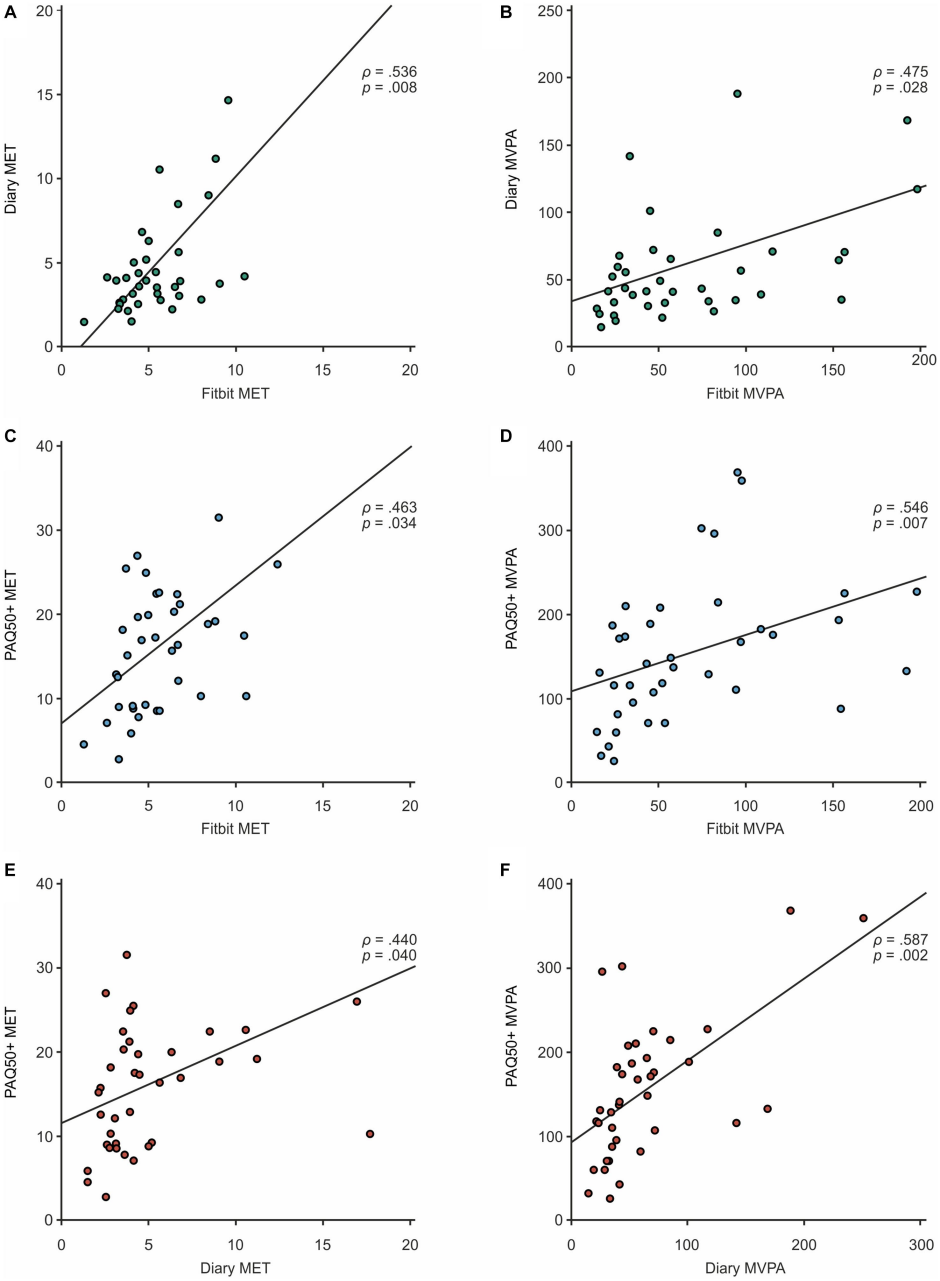
Correlation plot for comparison of MET and MVPA deriving from **(A,B)** Fitbit vs. diary (top), **(C,D)** Fitbit vs. PAQ50 (middle), and **(E,F)** diary vs. PAQ50+ (bottom).

In the correlation analysis, MET values of all three methods were moderately correlated (Fitbit vs. diary, *ρ* = 0.54, *p* = 0.01; Fitbit vs. PAQ50+, *ρ* = 0.46, *p* = 0.03; diary vs. PAQ50+, *ρ* = 0.44, *p* = 0.04) (Figure 1 and Supplementary Table S2). This effect was also reflected by the MVPA scores (Fitbit vs. diary, *ρ* = 0.48, *p* = 0.03; Fitbit vs. PAQ50+, *ρ* = 0.55, *p* = 0.01; diary vs. PAQ50+, *ρ* = 0.59, *p* = 0.01), pointing to a proportional association of these main parameters between all three methods. In line with the discrepancies in the descriptive analysis, a lower level of agreement was observed between diary and Fitbit subdivided moderate and vigorous activity scores (moderate intensity, *ρ* = 0.31, *p* = 0.18; vigorous intensity, *ρ* = 0.34, *p* = 0.18). Interestingly, activity calories were also highly correlated despite presenting strong differences in the comparison of absolute data (see again Supplementary Table S2).

**Figure 2:**
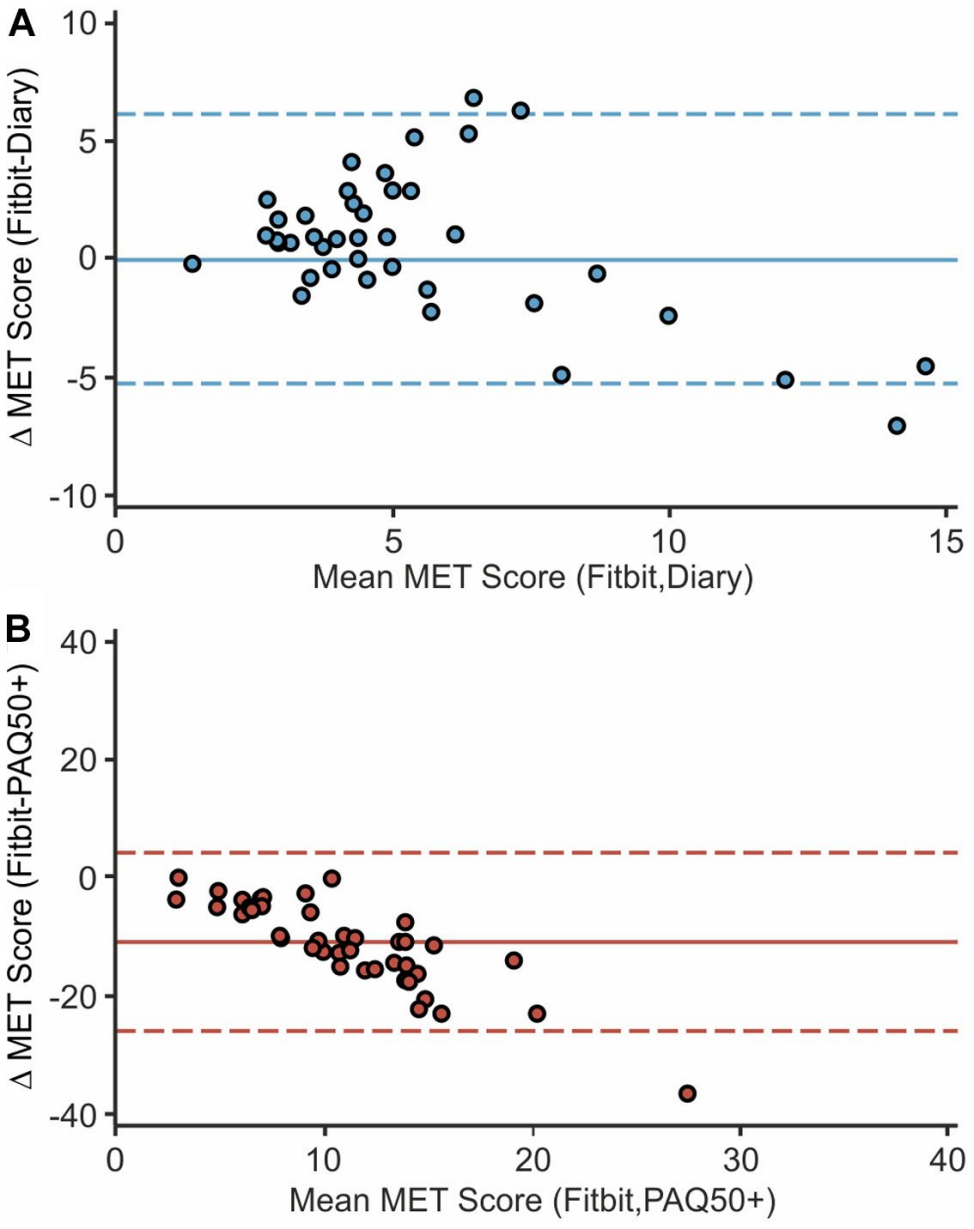
Bland-Altman-Plots of Fitbit and diary MET **(A)**, as well as Fitbit and PAQ50+ MET **(B)** are presented. Combined mean values on the x-axis are plotted against the difference in absolute values of the two instruments compared. The continuous horizontal line marks the mean difference; dashed lines indicate the difference's 95% limits of agreement defined as 1.96x standard deviation.

We furthermore illustrated the data in Bland–Altman-Plots to visualize the agreement between the subjective estimated MET deriving from the diary or questionnaire and the fitness tracker data based on each method’s total mean values (see Figure 2): In the plot for diary and Fitbit MET, an average difference between the two instruments MET_Fitbit-Diary_ close to zero (0.45 ± 2.90) suggested a good overlap among the absolute values. The majority of data points were randomly located around the mean line and within the tolerance range pointing to a measurement with very low bias. Only two data points were detected as minimal outliers, however one overestimating and the other one underestimating physical activity in the diary compared to the Fitbit, so that a random error can be assumed. The plot considering PAQ50+ and Fitbit MET however confirmed the previously raised assumption that physical activity in the questionnaire was overestimated. While the mean difference MET_Fitbit-PAQ50+_ already showed a negative deviation (−10.73 ± 7.67), almost all data points in the scatterplot were also located in the negative range leading to a structured error in the PAQ50+ data. When looking at the point cloud it is also striking that there seems to be a proportional bias with an increasing overestimation of physical activity at higher mean values. Again, one subject was detected being outside the 95% limits of agreement.

### Influence of confounding factors

As a further step, we aimed to identify confounding factors, which could have an impact on fitness and activity assessments. We therefore performed partial correlation analyses comparing the objective Fitbit MET and MVPA data to our diary and PAQ50+ data, controlling for conceivable factors such as cerebrospinal amyloid-β 1–42, amyloid-β 42/40-ratio, tau, phospho-tau, MoCA as well as HADS score and years of education. Corresponding zero-order correlations to check for associations between the tested and the correcting variables were also included to rule out non-conclusive deviations of the corrected analysis compared to the uncorrected correlation. Summarizing these results, MET and MVPA data of both the diary and the PAQ50+ appeared robust against the correction (see Table 3). The majority of corrected correlation coefficients presented a percentage difference lower than 0.1 compared to the uncorrected analysis. Only the partial correlation of diary MET corrected for tau (0.12), phospho-tau (0.13), as well as diary MVPA corrected for HADS (0.13) showed a stronger deviation >0.1. In all cases, there was (like for all other parameters) no significant zero order correlation between the physical activity parameters and the correcting variable (for tau: Fitbit MET, *ρ* = 0.09, *p* = 1, diary MET, *ρ* = −0.4, *p* = 0.07; for phospho-tau: Fitbit MET, *ρ* = 0.05, *p* = 1, diary MET, *ρ* = −0.38, *p* = 0.92; for HADS: Fitbit MVPA, *ρ* = −0.24, *p* = 1, diary MVPA, *ρ* = −0.02, *p* = 1) that could have indicated a causal relationship between these variables, and therefore supported the assumption of a systematic influence of the correcting variable in partial correlation analysis.

**Table 3:** Partial correlation analysis correcting for potential confounding factors.

Controlling variable	Fitbit MET vs. Diary MET	Fitbit MVPA vs. Diary MVPA	Fitbit MET vs. PAQ50+ MET	Fitbit MVPA vs. PAQ50+ MVPA
CSF Amyloid-β 1–42^a^	0.505^*^ (0.01)	0.400^*^ (0.00)	0.467^*^ (0.02)	0.526^*^ (0.00)
CSF Amyloid-β 42/40-ratio^b^	0.531^*^ (0.01)	0.405 (0.01)	0.495^*^ (0.00)	0.514^*^ (0.01)
CSF Tau^a^	0.601^**^ (0.12)	0.400 (0.02)	0.495^*^ (0.00)	0.519^*^ (0.00)
CSF Phospho-tau^a^	0.576^**^ (0.13)	0.385^*^ (0.04)	0.485^*^ (0.02)	0.517^*^ (0.01)
MoCA	0.502^*^ (0.06)	0.475^*^ (0.00)	0.428^*^ (0.08)	0.542^*^ (0.01)
HADS	0.539^**^ (0.01)	0.535^*^ (0.13)	0.464^*^ (0.00)	0.558^**^ (0.02)
Education years	0.517^*^ (0.04)	0.469^*^ (0.01)	0.442^*^ (0.05)	0.541^*^ (0.01)

a *n* = 35/38 participants.

b *n* = 34/38 participants.

Partial *ρ* correlation coefficients are indicated, together with the percentage difference from the uncorrected correlation analysis in brackets. ^*^indicates *p* < 0.05, ^**^indicates *p* < 0.01, respectively. CSF, cerebrospinal fluid; MoCA, Montreal Cognitive Assessment; HADS, Hospital Anxiety and Depression Scale; MET, metabolic equivalent of task; MVPA, moderate-to-vigorous physical activity (in minutes).

## Discussion

**Figure 3:**
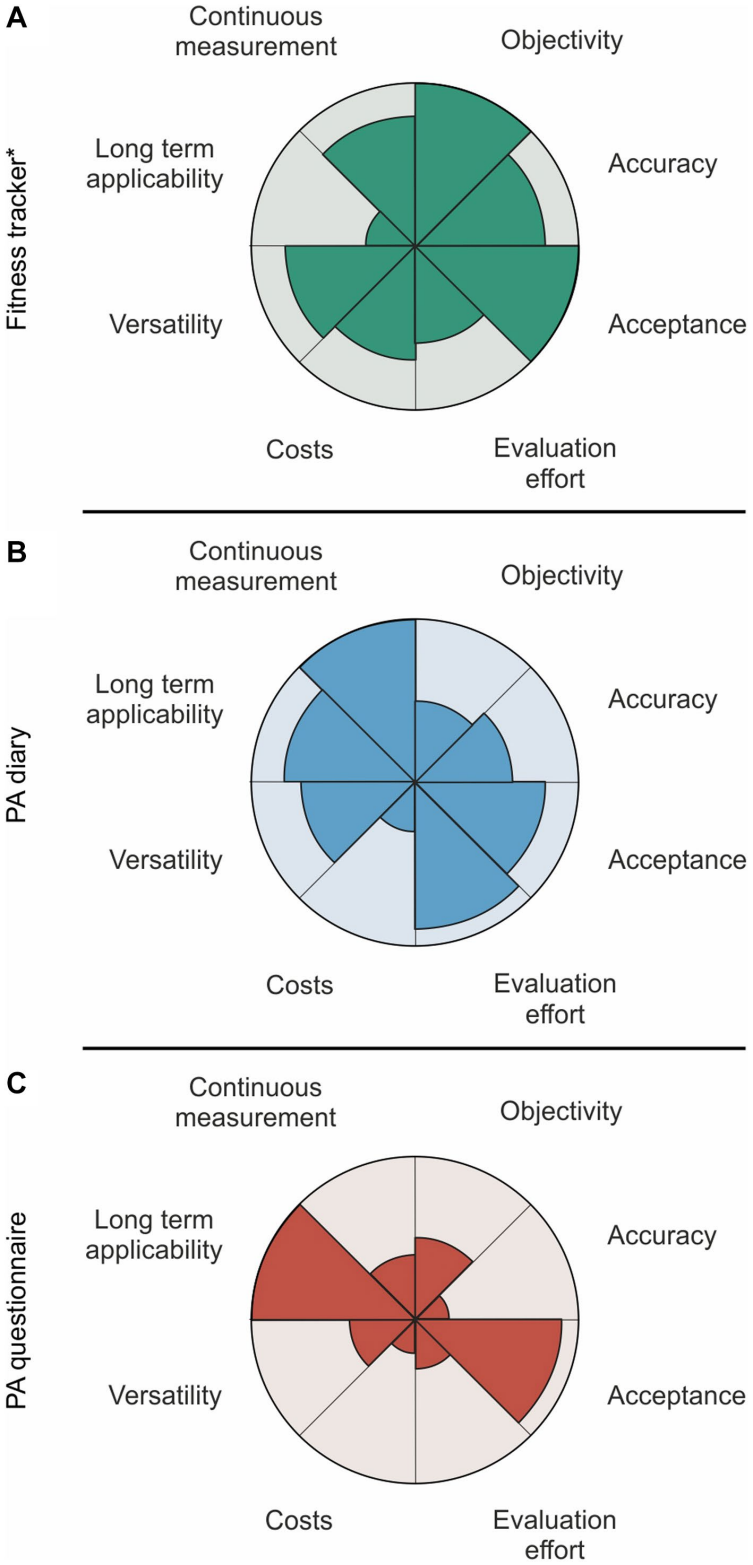
Visualized strengths and weaknesses of each method applied in a cohort with Alzheimer’s disease patients: **(A)** Fitness tracker, **(B)** PA diary, **(C)** PA questionnaire. ^*^An effective data acquisition via wearable tracker is strongly depending on the smartphone use for regular synchronization, which is still increasing in the elder generation (in Germany). PA, physical activity.

We here present three different methods of physical activity assessment in a population with AD during a 6 months intervention study (Dementia-MOVE): (1) wearable fitness trackers, (2) diaries on daily activities and (3) questionnaires on physical activity. We aimed at comparing these methods in terms of their feasibility and reliability when study participants are affected by AD and therefore suggest a homogenization method in the presented study. We show that different physical activity assessment methods can be well homogenized when paying attention to inter- as well as intra-individual differences. In this context, one of the strengths of our study is that our study group is well-characterized and relatively homogenous due to our rigorous inclusion and exclusion criteria, resulting in a comparatively smaller sample size that still yields robust results, also supported by our previous power calculations. Therefore, all physical activity assessment methods showed a high compliance and acceptance rate during our 6 months intervention. MET and MVPA scores from fitness trackers and diaries are highly overlapping, and their physical activity assessment independent of Alzheimer’s pathology and cognitive state. An overview of the advantages and disadvantages of the different assessment methods and limitations is illustrated in Figure 3, for which we summarized eight categories, which are important to consider when trying to find the most suitable method for activity assessment. We therefore assigned a score between zero and ten (with zero = lowest score, and ten = highest score) based on our experience and estimation and then visualized the scores proportionally. The different assessment methods will be discussed in detail highlighting their strengths and limitations in the following sections.

### The wearable tracker as a modern extension to physical activity monitoring in AD

Commercially available fitness trackers get more and more refined due to technological improvements, are meanwhile widely integrated into our daily life and are even used for health promotion and disease prevention (39–42). The reliability of wearable trackers has been evaluated before, pointing out a good validity of the devices’ accelerometric data such as steps, and covered distance but also the heart rate (43), whereas the calculation of activity minutes or calorie consumption are still discussed to give heterogeneous results especially in the elder generation (44, 45). The advantage of this tracker is that it is small, affordable, wearable as a watch and usable without the need of extensive habits or without being cognitively demanding and therefore independent of AD-pathology. This is also reflected by our proven 100% compliance in wearing fitness trackers as supported by the tracker data. Still, taking these previous publications into account, our main parameter for comparison was the MET score generated out of the Fitbit’s walking distance. We indeed registered deviating values for moderate and vigorous training intensity minutes when comparing Fitbit and diary data, however, the sum of both indicated as MVPA values were again strongly overlapping. This effect is presumably based on a differing distinction of physical activity intensity between both tools. Based on our results, the Fitbit had a lower threshold for classifying activities as vigorous intensity. It would be conceivable that the Fitbit has a higher sensitivity, as it is theoretically capable to incorporate not only MET values but also heart rate and personal data such as the age of the user into its analysis, yet Feehan et al. as well reported a tendency of the device to overestimate vigorous activity which is also reflected by our results (44). Further discrepancies were also detected when comparing the activity calories of all three different measuring tools, again pointing to a fundamentally divergent calculation algorithm. Unfortunately, we cannot definitively answer these questions, as most of the Fitbit’s detailed analysis algorithms, and threshold values are subject to company secrecy, which is one of the largest minus points. Therefore, the usage of the summarizing MVPA next to the step count in intervention studies is recommendable. Due to the inaccuracies stated above the activity calorie consumption however should be a parameter considered with lower priority.

Concerning the applicability of wearable devices in an elderly population, we stated challenges with synchronization which could be solved by narrowing the span of the synchronization process and by integrating the smartphones of the participants and their caregivers if available. This required a stronger involvement of the participants, which still led to the 100% compliance as mentioned before. In general, the experience in entrusting participants with these technical tasks under close instruction was positive, confirming the previously described large acceptability by older adults (46). Also worth mentioning is the high amount of additional information, such as sleep and vital parameters continuously measured by the device. However, the effort required for a continuous application for longer intervention periods, potentially taking months to years, would require a higher inclusion of the participants for regular synchronization and therefore a high availability of smartphones in this cohort, which is currently with more than 40% of smartphone users in the generation 65+ in Germany still in progress (47). With that in mind, an application of fitness trackers in long-term studies as an objective control can be a valuable option, especially when regular synchronization processes and closer follow-ups are performed. Yet, previous findings stated that also short measuring periods are sufficient to capture a ground physical activity level of older people, so even with short-term application, the information gain of wearable trackers can be valuable depending on the intervention design and overall objective (48). Furthermore, to our knowledge there has not been any previous intervention study in AD applying Fitbit wearable fitness trackers which we proved as feasible in the presented study.

### Subjective physical activity assessment via diary is feasible in (early) AD

We established the physical activity diary in our study protocol to continuously keep track of the participants’ dynamics in activity and to supervise protocol adherence over the course of the intervention. One of the main advantages of the diary is the flexibility in design, which allows a precise tailoring to the respective research question. Inaccuracies due to recall bias are reported for diaries, yet this source of error is minimized compared to questionnaires by entering activity information on a daily base (49). Integrating a physical activity diary in exercise study protocols also increased adherence in previous studies, which is underlined by a high compliance in our AD cohort, both in the intervention and the control group (50, 51).

When comparing the data captured in the diary with the fitness tracker output, we registered a surprisingly strong concordance of the MET and MVPA mean values, which was not only reflected when comparing data of the matching recording days, but also of the whole intervention period reinforcing the equally good suitability for assessing the ground physical activity level of both methods. The further correlation and largely bias-free visualization in the Bland–Altman-Plot confirmed a high agreement among diary and tracker.

When examining our data for possible AD-related influencing factors, that may have biased our assessment, we found no significant association. This is particularly interesting, since even the cognitive state reflected by the MoCA score did not influence the correlation between the different assessment tools. There is previous literature pointing to a possible association between tau accumulation and subjective physical activity assessment, which was not clearly reflected in our sample (52). This however has to be interpreted with caution, since CSF markers from our sample originated from the timepoint of diagnosis, which in most cases did not correspond with the intervention start (with CSF analysis performed in a range of a maximum time difference of 1954 days before intervention start to 189 days after intervention start). In this context, however, previous literature has pointed to only slowly progressing longitudinal changes in CSF markers in AD (53–55).

Especially as an addition to wearable trackers, the diary requires low effort and costs in the assessment period, making its use attractive for smaller intervention studies that cannot afford to supervise the tracker’s measurement for an extended period of time. Still, precise instructions and a regular check for quality and compliance is crucial to guarantee good results from this method. A combination of both tools, as performed in our study, is also helpful to enable a further verification of the subjective diary data in the individual study cohort.

### Structured questionnaires recommended under precise instructions

Based on their design, structured questionnaires can be a simple and short way to assess physical activity, which is why they are often applied especially in observational studies analyzing the influence of physical activity on cognitive health and AD disease progression (56, 57). Depending on the distinct questionnaire, they usually take only a few minutes to fill out and offer specified scoring routines that enable a fast and low-effort information gain (10). However, difficulties in assessing average physical activity details are well-described for short-term recall questionnaires, and seem to occur even more likely in people with cognitive impairment (12).

We stated an overestimation of physical activity by the study participants in the PAQ50+ compared to the Fitbit and the physical activity diary which was also reflected in the Bland–Altman-Plot, visualizing a negative difference of MET_Fitbit-PAQ50+_ values for almost all participants. There was also a proportional trend in the graphic presentation with an increasing extent of overestimation for participants with a higher physical activity pointing to a generally rather reduced accuracy of the questionnaire. These results are supported by previous findings, showing that people with a higher activity level are more likely to over-report their physical activity (58). Furthermore, questionnaires are considered to be especially applicable for reporting vigorous activity, which can, in combination with memory recall problems, lead to an overestimation of physical activity (59–61). The design of the PAQ50+ to estimate the average weekly duration of different activities based on the last month was possibly challenging for many participants in our cohort due to their cognitive impairment. Eventually, a simplified design concerning the questionnaire’s reference time-period (e.g., daily activity) might be more suitable for people having a hard time to remember their retrospective activities in intervention studies (62).

Concerning our correlation analysis, there was still an association of the PAQ50+ with the fitness tracker and diary assessment, which might indicate that the information entered in the questionnaire still follows a constant pattern with preserved proportionality despite the fundamental differences in absolute values, possibly enabling an examination of intra-individual physical activity dynamics. However, this alone is not sufficient to consider the PAQ50+ measurement reliable, at least based on the results of our cohort.

Altogether, the limited application of questionnaires in AD intervention studies should be considered, and the precise research question and the suitability of the questionnaire for the specific cohort need to be discussed. Special attention should be paid to understandable and simple instructions, when selecting an appropriate questionnaire for participants with cognitive impairment.

### How to choose the right physical activity monitoring method and parameters in AD?

The decision on the assessment method for physical activity is *a priori* dependent on the objective and the study sample itself. We deliberately do not define a gold standard for our analysis since all three methods have different advantages and disadvantages: Fitness trackers can be used independently of the cognitive state of the user and are well accepted by the study participants. However, synchronization of data and therefore availability of corresponding tools, especially smart phones in elder generations need to be considered. In the absence of a smartphone, the collection and synchronization by the study team on a regular base can be related to a higher effort, whereas continuous synchronization assured by the participants’ smartphones can enable longer and continuous periods of data collection, which has to be considered as a caveat in the planning phase of an intervention study.

For physical activity diaries, participants with AD need to be in a cognitive state comparable to our participants. Physical activity questionnaires can entail risks of overestimating physical activity due to insufficient recall on retrospective activity. Concluding from our results, fitness trackers and physical activity diaries are both feasible and recommendable, leading to a highly comparable estimation of physical activity in AD pathology and can be homogenized and compared as described above via calculation of MET and MVPA. As assessed parameters, it is recommendable to focus on MET, MVPA and on accelerometric data such as step count, and less on activity calories and different activity intensities which are less robust since these are dependent on different variables. Altogether, multimodal assessment of physical activity with a focus on most robust and replicable parameters can lead to an increased quality of exercise intervention studies in AD pathology and even other neurodegenerative diseases entailing cognitive decline, helping to gradually close the knowledge gap on how physical activity and (cardiorespiratory) fitness influence each other in the elder generation and how they mutually impact disease progression.

## Data availability statement

The data supporting the conclusions of this article will be made available on reasonable request.

## Ethics statement

The studies involving humans were approved by ethics committee of the University Hospital RWTH Aachen (EK 306/18). The studies were conducted in accordance with the local legislation and institutional requirements. The participants provided their written informed consent to participate in this study.

## Author contributions

AH, AC, and KR contributed to the conception and design of the study. AH supervised the study and the methodology. CH developed the database and performed the data management. SD performed the statistical analysis and wrote the first draft of the manuscript. SD, LH, and JP performed data acquisition. AK and his team supervised the sports program. All authors corrected and reviewed the article and approved the submitted version.

## Footnotes

^1^ https://help.fitbit.com/articles/en_US/Help_article/1379.htm

^2^ https://www.python.org

^3^ https://www.ibm.com/products/spss-statistics
