# Supplementary material for: Physical activity monitoring in Alzheimer’s disease during sport interventions: a multi-methodological perspective
Source: Front Neurol. 2023 Sep 22;14:1195694. doi: 10.3389/fneur.2023.1195694 (PMC10557074; doi:10.3389/fneur.2023.1195694)
Supplement: Supplementary file 1 [file Data_Sheet_1.docx]

# Supplementary Table 1. Exemplary PA diary page translated into English language.

Intervention Week: _______ Calendar Week: _______ Name: ______________________________________________

|  | xx/xx/xx | xx/xx/xx | xx/xx/xx | xx/xx/xx | xx/xx/xx | xx/xx/xx | xx/xx/xx |
| --- | --- | --- | --- | --- | --- | --- | --- |
|  | Monday | Tuesday | Wednesday | Thursday | Friday | Saturday | Sunday |
| *Supervised Exercise** |  |  |  |  |  |  |  |
| Home Sports – *30min 1x/week**  (Which activity? How long?) |  |  |  |  |  |  |  |
| *Training Cards – 15min 2x/week*  *(Which card / exercise?)** |  |  |  |  |  |  |  |
| Complaints during exercise |  |  |  |  |  |  |  |
| Perceived exertion during physical activity according to the Borg scale (0-10 points) |  |  |  |  |  |  |  |
| Sleep (hours per night) |  |  |  |  |  |  |  |
| Medication taken on demand |  |  |  |  |  |  |  |
| Weight (1x/week without clothes) |  |  |  |  |  |  |  |

Other comments: __________________________________________________________________________________________________________________________________________________________________________________________________________________________________

*Information presented in italic letters were only included in the PA diary for participants assigned to the intervention group

**Supplementary Table 2. Spearman’s rank correlation analysis of the three methods applied.**

| *Variable* | *Fitbit vs. Diary* | | *Fitbit vs. PAQ50+* | *Diary vs. PAQ50+* |
| --- | --- | --- | --- | --- |
|  | *record period* | *intervention period* |  |  |
| MET | .529**  [.253; .740] | .536**  [.230; .763] | .463*  [.185; .685] | .440*  [.106; .696] |
| Moderate Activity (min) | .155  [-.194, .462] | .309  [-.015, .582] | .515*  [.223, .740] | .537**  [.235, .753] |
| Vigorous Activity (min) | .130  [-.191; .429] | .340  [.001, .621] | .372  [.123, .565] | .556**  [.310, .730] |
| MVPA (min) | .333  [-.023, .609] | .475*  [.165, .692] | .546**  [.282, .748] | .587**  [.321, .776] |
| Activity Calories (kcal) | .462*  [.156; .689] | .505*  [.198, .725] | .459*  [.123, .707] | .485*  [.201, .703] |

Spearman’s *ρ* correlation coefficients are presented. 95% confidence intervals are computed via BCa-bootstrapping with 2000 samples. *indicates *p* <0.05, **indicates *p* <0.01. Correlation of Fitbit and diary data was carried out referring to means of recorded days and their matching diary entries only as well as means of the total intervention period.
